# Selection of Predatory Mites for the Biological Control of Potato Tuber Moth in Stored Potatoes

**DOI:** 10.3390/insects11030196

**Published:** 2020-03-20

**Authors:** Juan R. Gallego, Otto Caicedo, Manuel Gamez, Joaquin Hernandez, Tomas Cabello

**Affiliations:** Research Centre for Mediterranean Intensive Agrosystems and Agrifood Biotechnology (CIAMBITAL), Agrifood Campus of International Excellence (CEIA3), University of Almeria, Ctra. de Sacramento, s/n, 04120 La Cañada, Almeria, Spain; jgg436@ual.es (J.R.G.); ocs90@outlook.com (O.C.); mgamez@ual.es (M.G.); jhrodri@ual.es (J.H.)

**Keywords:** *Phthorimaea operculella*, *Blattisocius tarsalis*, *Macrocheles robustulus*, prey acceptance, functional response

## Abstract

Worldwide, the potato tuber moth (PTM), *Phthorimaea operculella* (Zeller), is one of the most severe pests affecting potato (*Solanum tuberosum* L.), whether in open-air crops or during tuber storage. This work examines the potential control of this pest by two species of predatory mites, *Macrocheles robustulus* (Berlese) and *Blattisocius tarsalis* (Berlese), on pest eggs under laboratory conditions. In the two first assays, the acceptance rate of the pest eggs was assessed for each predatory mite. Then, in a third assay, the functional response of *B. tarsalis* was studied. The results showed that *Macrocheles* *robustulus* did not prey on the pest eggs (number of eggs surviving = 4.33 ± 0.38), whereas *B. tarsalis* did (number of eggs surviving = 0.5 ± 0.5). Likewise, *B. tarsalis* showed a type II functional response when it killed the eggs. The results showed the potential use of *Blattisocius tarsalis* as a biological control agent of *P. operculella* in potato under storage conditions.

## 1. Introduction

Potato (*Solanum tuberosum* L.) is the fourth major food production crop in the world, after wheat, maize and rice. The cultivation area exceeds more than 19 million hectares and production is higher than 388 million tonnes [1]. Pests and diseases cause pronounced losses in potato crops; on average, 40.3% of losses are caused by plant pathogens and viruses, 21.1% by animal pests and 8.3% by weeds [2]. In the second group, the principal arthropod pests affecting this crop are *Leptinotarsa decemlineata* (Say) (Coleoptera: Chrysomelidae), the potato tubermoth complex (Lep.: Gelechiidae) and aphids, mainly *Myzus* (*Nectarosiphon*) *persicae* Sulzer (Hemiptera: Aphididae), which are not just a pest but also an important virus vector in the crop [2,3]. Among the potato tubermoth complex is the common potato tubermoth (PTM), *Phthorimaea operculella* (Zeller), the Andean potato tubermoth, *Symmetrischema tangolias* (Gyen), and the Guatemalan potato tubermoth, *Tecia solanivora* Povolný [4], all economically important pest species. Of these, the PTM is a global pest of solanaceous crops and weeds, and is especially devastating to potatoes [5], causing losses of up to 80% in the field and up to 100% during storage [6,7,8,9].

PTM is responsible for the greatest economic losses in potato. The pest originally comes from the tropical mountainous regions of South America. Currently, its distribution is considered cosmopolitan because it has expanded across five continents and is present in more than 90 countries [4,10]. The economic damage is associated with the close relationship between PTM and its host, its high adaptability to daily and seasonal changes, its high reproductive potential, and its capacity to develop on other Solanaceae, whether cultivated as wild [4,6,10].

*P. operculella* attacks all the vegetative parts of the plant causing mines due to larvae feeding. When the food value of the leaves decreases, the larva moves from the aerial part of the plant to look for tubers [4]. This movement usually happens immediately before the harvest. The pattern of the damage evolution caused by *P. operculella* means that control must be carried out in the field as well as under storage conditions [11]. For more than a century, chemical control has been widely used for pest control in potato production [12]. However, in response to potential health risks, consumer demand for healthier products, long-term environmental costs and the development of resistances to chemical pesticides, the popularity of integrated pest management (IPM) has increased as an alternative to chemical control [13,14,15,16]. Legislation reflects this shift with policies introduced to reduce their use and restrict the active ingredients of certain insecticides [16,17]. For example, all EU member states adopted IPM policies in 2014 as the main strategy for reducing the negative impacts caused by rapidly withdrawing pesticides from food production [16,18]. Furthermore, it has been suggested that IPM is the only way of controlling moths present in the stored tubers [19]. Therefore, there is a growing interest in using biological control tactics.

The use of predatory mites, particularly those belonging to Phytoseiidae family (Acari: Mesostigmata), has become one of the main IPM tools to protect crops worldwide [16,20,21]. The use of other families of predatory mites for the control of edaphic plant-eaters is also common and these are available commercially. Most of the available species belong to the Macrochelidae and Laelapidae families [21,22]. Nevertheless, there are other species, which belong to the same family or to other families, that have a potential practical application but have not yet been studied properly [22].

Several studies have considered the use of species belonging to the Blattisociidae family as a potential control agent of mites and pest insects under storage conditions [23,24,25], although little knowledge exists about the biology of many species in this genus [26]. In some laboratory studies, predation by *Blattisocius tarsalis* (Berlese), *B. dendriticus* Berlese and *B. keegani* Fox has been researched for their potential as biological control agents [23,26,27]. Recently, the potential of *B. mali* (Oudemans) in control *P. operculella* has been studied with promising results [25].

Another group that includes abundant predatory mites is the Macrochelidae family in the order Mesostigmata; these species have also proven to be important biological control agents in Diptera species and other pest insects [15,28,29].

Considering the above information, this work aims to assess the potential of *B. tarsalis* and *Macrochels robustulus* (Berlese) mites as biological control agents of PTM. To this end, two acceptance assays of PTM eggs, as *B. tarsalis* and *M. robustulus* food have been conducted along with a further assay in which *B. tarsalis* predation behaviour has been analysed in terms of changes in prey density (the PTM eggs), studying the functional response.

## 2. Materials and Methods

### 2.1. Biological Material and Experimental Conditions

The *B. tarsalis* mites were identified and obtained from a casual infestation of an *Ephestia kuehniella* Zeller (Lepidoptera: Pyralidae) small moth in the Agricultural Entomology Laboratory of the University of Almeria in September 2018, using the Nesbitt [30] and Haines [31] keys. The specimens were reared in the Agricultural Entomology Laboratory of the University of Almeria for 9 months before the experiments began. The *B. tarsalis* mite colonies were kept in the laboratory following the Nielsen et al. methodology [24] with slight modifications. Plastic containers were used to house them; these were filled with a vermiculite layer, which had a relative humidity (R.H.) of 75%, obtained from a saturated aqueous solution of NaCl [32]. The mites were fed with 0.20 gr. of *E. kuehniella* eggs every 3 days. The containers were kept under the following environmental conditions: 25 ± 1 °C and 16:8 light:darkness (L:D) hours. Koppert Biological Systems (La Mojonera, Almeria, Spain) provided cardboard cylinders of 50,000 *M. robustulus* predatory mites (Macro-mite^®^).

The PTM population was reared in the same laboratory after the specimens were provided by the Plant Protection Laboratory of Almeria (Andalusian Regional Government, Spain). The rearing methodology described by Fenemore [33] was used, and small potatoes (variety: Marilyn^®^, category 1, size 28/45 mm; H.Z.P.C. Holland B.V., Joure, The Netherlands) were used to feed the larvae. Plastic containers (1000 mL) were used as mating and oviposition chambers for the PTM with 20 adult pairs confined inside each one. The containers were closed at the top with surgical gauze and secured with an elastic band. A filter paper disc was placed over the gauze as a substrate for the oviposition of the females. The filter paper discs carrying the eggs were placed in contact with the tubers, the surfaces of which were previously prepared with holes made with a pin to facilitate entry of the hatching larvae into the tubers. Additionally, a vermiculite layer was arranged to promote the pupal formation. Once larval development was complete, the substrate was sieved to remove the pupae and place them into a new mating and ovipositional container until the adults emerged. The environmental conditions for the offspring were 25 ± 1 °C, 60–80% R.H. and a photoperiod of 16:8 h (L:D).

### 2.2. Acceptance Tests of P. operculella Eggs as Prey

Two “no-choice” bioassay tests were carried out in which acceptance and predation of PTM eggs by *M. robustulus* and *B. tarsalis* was assessed. To this end, the methodology of Gallego et al. was followed [25], with female mites placed individually in glass test tubes (7.0 cm × 1.0 of diameter). A piece of white cardboard (5.0 cm × 0.9 cm) was placed in each test tube to which five PTM eggs were stuck using a thin paintbrush (00), water was then introduced together with a piece of moistened sponge (0.5 × 0.5 cm). The test tubes were sealed with cotton. During the next 48 h, under the previously stated environmental conditions, the females were left to prey on the eggs. In the check (control), the process was carried out as above but without introducing adult female mites into the test tubes. 

The experimental design was univariate and fully randomised, with only one factor: predatory mite compared with check. There were 20 mite repetitions and 20 for the control repetitions. In the case of *M. robustulus* species, the experiment was repeated twice with different commercial batches. At the end of the assay, the eggs were examined under a binocular microscope and the number of eggs preyed upon and/or partially consumed by mites was counted. The eggs were then left to develop over the next 7 days to allow for possible PTM larvae emergence. The environmental conditions were 25 ± 1 °C, 80–90% R.H. and 16:8 h of L:D.

The values corresponding to the number of PTM eggs that survived were analyzed statistically using a generalized linear model (GZLM) with the Poisson distribution and the log link function; likewise, the average values were compared by pairs using the Wald test at *p* = 0.05. To perform this, IBM SPSS version 25 statistical software was used.

The mites’ effectiveness at controlling the PTM eggs was assessed using the modified Abbot equation [34]):

(1) $$ER=\left(\frac{M-M^{\prime }}{100-M^{\prime }}\right)*100$$

where, *ER* = efficiency rate (correcting % efficacy for the natural mortality), *M* = mortality rate in the treatment (mite) and *M’* = mortality rate in the check (control).

### 2.3. Study of Predation Behaviour at Different Prey Densities: Functional Response

The methodology of Nielsen [35] and Garcia-Martin et al. [36] was followed. The procedure was carried out after the prey acceptance bioassay, with the following exceptions: First, different PTM egg densities were offered to the female adult mite (1, 2, 3, 6, 9, and 12 PTM eggs); second, the exposure time to predation was only 24 h (1 day); and third, the number of treatment replications was 20.

Two statistical analyses were performed to fit the type of functional response to the mortality data collected. In the first one of these, a previous estimate was carried out to determine the type of functional response. The data were fitted to the polynomial function used by Juliano [37]:

(2) $$\frac{N_{e}}{N_{0}}=\frac{exp\left(P_{0}+P_{1}N_{0}+P_{2}N_{0}^{2}+P_{3}N_{0}^{3}\right)}{1+exp\left(P_{0}+P_{1}N_{0}+P_{2}N_{0}^{2}+P_{3}N_{0}^{3}\right)}$$

where *N_e_* is the number of prey eaten; *N_0_* the initial value of prey offered; and *P_0_*, *P_1_*, *P_2_*, and *P_3_* are the intercept, linear, quadratic, and cubic coefficients, respectively, estimated using the maximum likelihood method. Statgraphics version 18 software was used for the adjustments. *P_0_*–*P_3_* parameters were obtained from a logistic regression. If the *P_1_* coefficient was not significantly different from zero, it corresponded to a type I functional response; if the *P_1_* value was significantly negative, this would demonstrate type II functional response while a significantly positive *P_1_* value would demonstrate a type III functional response. We understand a value to be different from zero when zero is not included in its confidence interval.

A more exact statistical analysis was then conducted, and mortality data were fitted to the equations proposed by Hassell [38] (Equations (3) and (4)) and Cabello et al. [39] (Equation (5)) for predators (when there is no prey replacement):

Type I:

(3) $$N_{a}=N\left(1-EXP(-a’P\left(T-T_{h}\frac{N_{a}}{P}\right)\right)$$

Type II:

(4) $$N_{a}=N\left(1-EXP(-a’P\left(T-T_{h}\frac{N_{a}}{P}\right)\right)$$

Type III:

(5) $$N_{a}=N\left\{1-exp\left[-\frac{\alpha \cdot N\cdot P}{1+T_{h}\left(exp(-\alpha \right)-1)\cdot N}\left(T-T_{h}\frac{N_{a}}{P}\right)\right]\right\}$$

where *N_a_* represents the number of prey attacked, *N* the number of prey offered, *a’* the attack rate of the predator, *T* the time length of the assay, *P* the number of predators used, *T_h_* the handling time of the prey by the predator (capture time and feeding time), and *α* the predation potential. In this assay, the following data were used: *T* = 1 day and *p* = 1 predator. The statistical software used for this type of functional response fitting was TableCurve 2D, version 5.01.

## 3. Results

### 3.1. Acceptance Tests of P. operculella Eggs as Prey

**Figure 1:**
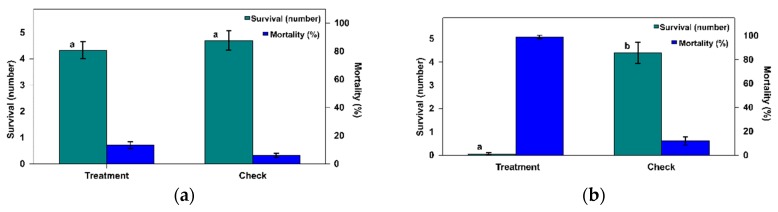
Number (±SE) of survivors and the mortality rate of *Phthorimaea operculella* eggs when exposed for 48 h to an adult female mite of (**a**) *Macrocheles robustulus* or (**b**) *Blattisocius tarsalis* compared to the check, in the acceptance of prey assay test under laboratory conditions (values with different letters mean significant differences at *p* = 0.05).

For the predatory species *M. robustulus*, the average number of surviving PTM eggs was 4.33 ± 0.38 with no differences when compared with the check 4.70 ± 0.49 (Figure 1a). In the statistical analysis of the data (the number of surviving eggs), the treatment had no effect (Omnibus test: likelihood ratio chi-squared test = 0.358, d.f. = 1, *p* = 0.549). Likewise, the corresponding mortality values were not dissimilar: 13.33 ± 2.93% and 6.00 ± 2.10, respectively, for the mite and the check. 

In contrast, the number of surviving PTM eggs and the mortality rate for adult females *B. tarsalis* mites was different to that of the check (Figure 1b). In the statistical analysis of the number of survivors, a highly significant effect was found (Omnibus test: likelihood ratio chi-squared test = 112.414, d.f. = 1, *p* < 0.0001). The number of surviving eggs in the mite treatment (0.05 ± 0.05) was significantly lower than in the check (4.40 ± 0.47). Such values represent a mortality rate of 99.00 ± 1.00% in the treatment and 12.00 ± 3.37% in the check. As a result, according to Equation (1), the efficiency rate *ER* = 98.86%.

### 3.2. Study of Predation Behaviour at Different Prey Densities: Functional Response

Table 1 shows the adjustment parameters to the polynomial function (Equation (2)) of the number of PTM eggs preyed upon by female *B. tarsalis* mites. As mentioned before, in the Materials and Methods section, the *P_1_* value was significantly negative; therefore, the above analysis demonstrates that this mite presents a type II functional response (when considering a value different from zero, when zero is not included in its confidence interval, as happened in this case).

**Figure 2:**
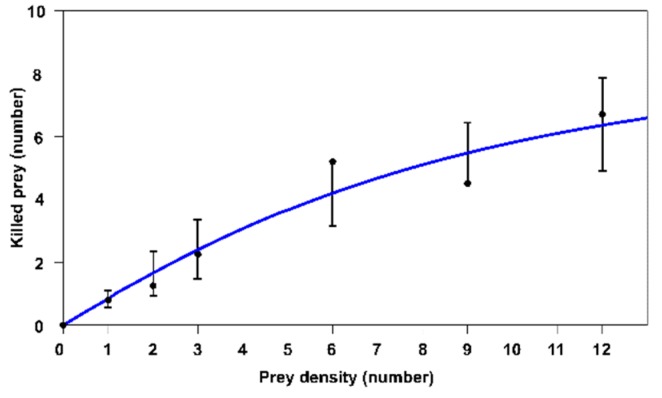
Type II functional response (number of pest eggs killed) of the adult female of *Blattisocius tarsalis* when different egg densities of *Phthorimaea operculella* were offered as prey for 24-hours under laboratory conditions (whisker plot: 95% confidence intervals).

The above result is consistent with the fitting results carried out with the three types of functional responses shown in Table 2. These confirm type II (Equation (4)), because the corrected Akaike Information Criterion (*AIC_C_*) shows the lowest value. Figure 2 presents the type II functional response of the predatory mite.

## 4. Discussion

With the aim of selecting a predatory mite species for the biological control of *P. operculella*, the results of this study have demonstrated the potential of *M. robustulus* and *B. tarsalis*. The first acceptance tests allowed us to select *B. tarsalis* in the functional response study against the pest.

Since 2010, the *M. robustulus* species has been commercially available to use against Diptera, Thysanoptera and Lepidoptera pests [21,40]. This species is effective at controlling thrips in soil [41,42]. The available information, on this species and others belonging to the same group, is poorly researched and studied. However, they can be useful biological control agents against organisms that live all or part of their lives in the soil [40]. Given the control potential of this species as indicated above, we performed acceptance tests of PTM eggs as prey for *M. robustulus*.

However, the results seem to indicate that PTM eggs are not adequate prey for *M*. *robustulus* even though the Macrochelidae family is characterised by its acceptance of a wide prey range. A possible explanation might be that this family is closely linked to the habitat. Accordingly, Filipponi [43] found that densities of several *Macrocheles* species depended on the composition and physical conditions of the environment. This author also observed that in each of the mite’s development stages, it has a different prey preference. In this way, deutonymphs and protonymphs prefer to attack eggs whereas the adults prefer dipteran larvae. In part, this could explain the poor results obtained. Firstly, the assay was performed in a test tube, which is very different from the preferred habitats. Secondly, the test we carried out using only adult female predators, whose prey stage preference appears to be larvae. This has been cited by Filipponi for at least 21 species of Macrochelidae [43]. Nevertheless, other species of the same family (e.g., *M. muscaedomesticae* (Scopoli)) have been observed preying on eggs and small larvae from this species [44]. 

The differences found between *M. robustulus* and *M. muscaedomesticae* may be due, on the one hand, to the above-mentioned differences in prey preference between species and stages; on the other hand, it might be due to the different methodology followed in the trials. In this respect, *M. muscaedomesticae* is the most studied species of this genus. The species has demonstrated effective control of the common fly, *Musca domestica* L. (Diptera: Muscidae) and other synanthropic dipteran species [28]. Nevertheless, in this case, effectiveness requires the combined presence of other alternative prey, such as mites, nematodes, and annelids [45]. Available information on this species, and others belonging to the same group, is limited and poorly researched. However, they can be useful biological control agents against organisms that live all or part of their lives in the soil [40].

With respect to *B. tarsalis* and according to the consulted literature, this is the first time that PTM eggs have been cited as prey for this mite species. It should be mentioned that other species of the same genus such as *B. keegani* and *B. mali* have been cited as predators of PTM eggs [25,46,47].

According to the values for the surviving PTM eggs found, the predation rate of the *B. tarsalis* female adult averages 4.95 eggs/female in 48 h (99.00 ± 4.47% mortality) (Figure 1b), which represents a predation rate (over 24 h) of 2.24 eggs/female per day. This value is higher than the values found for *B. tarsalis* in *Plodia interpunctella* Hübner eggs (Lepidoptera: Pyralidae) [48] and in *E. kuehniella* eggs [24], or for *B. keegani* in *Amyelois transitella* eggs (Lepidoptera: Pyralidae) [26]. Although, *B. keegani* has been indicated as a possible alternative to *B. tarsalis* for the biological control of *E. kuehniella* in stored products [26]; therefore, it could be very interesting to test this species, in the future, in the control of *P. operculella* in storage potatoes.

Furthermore, our found values are similar to those found for *B. tarsalis* in *Ephestia cautella* eggs (Lepidoptera: Pyralidae) [23,24] and in other insect eggs [49]. With respect to PTM, *B. tarsalis* causes a mortality rate higher than those caused by *B. mali* (another species from the same genus) when dealing with the same amount of prey (PTM eggs) examined under similar conditions [25].

Of these species, *B. tarsalis* is the best studied [22,26]. *B. tarsalis* is a common predator of moth eggs (Lepidoptera), whose distribution is cosmopolitan in nature and has been reported across many regions of the world 23].

The predator *B. tarsalis* completes its development not only preying on the eggs of moths, *P. interpunctella* [48], *E. cautella* and *E. kuehniella* [23,24] but also eggs of other insect orders, such as mites, booklice and beetles [49,50]. Moreover, it has been mentioned as a phoront in some Lepidoptera species; Treat reported it on *Epizeuxis aemula* Hübner (Lepidoptera: Erebidae) and on *Apamea devastator* (Brace) (Lepidoptera: Noctuidae) [51].

*B. tarsalis* prefers lepidopteran eggs rather than eggs from other species. Haines observed this preference with respect to *Tribolium castaneum* Herbst beetle eggs (Coleoptera: Tenebrionidae) [23]. They can survive on them even when there is a lack of moth eggs, but they do not control them [15]. Additionally, it has been shown that this species can be an effective pest control agent in flour silos and stores, and has the capacity to control several pests that develop under storage conditions; such as the Mediterranean flour moth, *E. kuehniella* in flour silos [24,35,49,52], and the flour mite, *Acarus siro* L. (Acari: Acaridae), under laboratory conditions [53].

*B. tarsalis* shows a type II functional response (Table 1 and Table 2, Figure 2). This type of functional response is the most common for predatory mite and insect species [54]. Likewise, Riudavets et al. [49] found this type of response for the same mite species, but in two different prey eggs: *Lasioderma serricorne* (Coleoptera: Ptinidae) and *P. interpunctella*.

A functional response of this type is determined by two biological parameters: the attack rate of the predator (*a’*) and the handling time by the prey of the predator (*T_h_*). In our case, an attack rate on the PTM eggs of *a’* = 2.1258 ± 1.2096 (days^−1^) was found to have a better value than those reported by Riudavets et al. [49] for *L. serricorne* (0.043 days^−1^) and *P. interpunctella* (0.074 days^−1^), indicating that *B. tarsalis* seems to be a better control agent in *P. operculella* than the other two species.

This type of functional response allows us to determine the minimum release doses (corresponding to the optimal laboratory conditions) of the predatory mite under real conditions for biological control programmes. It should be mentioned that the potential of *B. tarsalis* as a biological control agent needs to be determined with further assays, whether under field conditions or in stored potatoes, because the *P. operculella* pest species develop in both situations.

## 5. Conclusions

1. The predatory mite *Blattisoicius tarsalis* accepted the eggs of the pest species *Phthorimaea operculella* as prey. In addition, the adult female has an efficiency rate of 98.86% in 48 h on the pest eggs.

2. The *Blattisocius tarsalis* mite presents a type II functional response, which is the most frequent found in predatory mite and insect species. The attack rate (*a’*) was 2.126 day^−1^ and the handling time of the prey (*T_h_*) was 0.101 days.

3. The potential of *Blattisocius tarsalis* as a potato tuber moth control agent, especially in stored potatoes, appears to be very good.

4. The development of biological pest control using *Blattisocius tarsalis* should evaluate, in trials on stored potatoes, the different methods utilising predatory mites developed to date: preventive methods (the use of non-prey food and/ or factitious prey), curative methods (augmentation by means of inoculative releases), etc.

## Figures and Tables

**Table 1:** Logistic regression analyses results for the proportion of *Phthorimaeae operculella* eggs killed by the adult female of *Blattisocius tarsalis* in the bioassay carried out under laboratory conditions.

Parameter	Value	SE	95% Confidence Level		F.R. Type
*P_0_* (Intercept)	1.3021	0.4021	0.5139	2.0902	II
*P_1_* (Linear)	−0.2499	0.1187	−0.4825	−0.0073	

**Table 2:** Parameters and statistical significance for the functional response equations type I, II, and III when different densities of *Phthorimaeae operculella* eggs were exposed to the adult female of *Blattisocius tarsalis*, for 24 h, under laboratory conditions.

Type	Fit Curve Parameters (±SE)			Statistical Parameters		
	*a’* (day^−1^)	*T_h_* (day)	*α*	d.f.	*R* ^2^	*AIC_C_*
I	0.8907 (±0.1175)	–	–	6	0.8996	0.3989
II	2.1258 (±1.2096)	0.1014 (± 0.0419)	–	5	0.9408	0.3106*
III	–	0.1678 (± 0.0193)	0.6339 (± 0.2725)	5	0.9393	0.3933

## References

[1] FAOSTAT Food and Agriculture Organization of the United Nations. FAO Statistics Division 2019. http://www.fao.org/faostat/en/#data/QC.

[2] Oerke E.C. (2006). Crop losses to pests. J. Agric. Sci..

[3] Radcliffe E.B. (1982). Insect pests of potato. Annu. Rev. Entomol..

[4] Kroschel J., Schaub B., Giordanengo P., Vincent C., Alyokhin A. (2013). Biology and ecology of potato. Insect Pests of Potato: Global Perspectives on Biology and Management.

[5] Das G.P., Raman K.V. (1994). Alternate hosts of the potato tuber moth, *Phthorimaea operculella*. Crop Prot..

[6] Rondon S.I. (2010). The potato tuberworm: A literature review of its biology, ecology, and control. Am. J. Potato Res..

[7] Trivedi T.P., Rajagopal D. (1992). Distribution, biology, ecology and management of potato tuber moth, *Phthorimaea operculella* (Lep.: Gelechiidae): A review. Int. J. Pest Manag..

[8] Hanafi A. (2007). The canon of potato science: 17. Arthropods/insects. Potato Res..

[9] Aryal S., Jung C. (2015). IPM tactics of potato tuber moth, *Phthorimaea operculella* (Lep.: Gelechiidae): Literature study. Korean J. Soil Zool..

[10] EPPO European and Mediterranean Plant Protection Organization. EPPO Global Database. https://gd.eppo.int/taxon/PHTOOP/distribution.

[11] Kroschel J., Canedo V., Wale S., Platt H.W., Cattlin N. (2008). Phthorimaea operculella (syn. *Gnorimoschema operculella*): Potato tuber moth. Diseases, Pests and Disorders of Potatoes: A (Colour) Handbook.

[12] Alyokhin A., Chen Y.H., Udalov M., Benkovskaya G., Lindstrom L., Giordanengo P., Vincent C., Alyokhin A. (2013). Evolutionary considerations in potato pest management. Insect Pests of Potato: Global Perspectives on Biology and Management.

[13] Fuglie K., Salah H.B., Essamet M., Temime A.B., Rahmouni A. (1993). The development and adoption of integrated pest management of the potato tuber moth, *Phthorimaea operculella*, in Tunisia. Int. J. Trop. Insect Sci..

[14] Dent D. (2000). Insect Pest Management.

[15] Gerson U., Smiley R.L., Ochoa R. (2008). Mites (Acari) for Pest Control.

[16] Vila E., Cabello T., Torres I., Guevara R. (2014). Biosystems engineering applied to greenhouse pest control. Biosystems Engineering: Biofactories for Food Production in the XXI Century.

[17] Lefebvre M., Langrell S.R., Gomez-y-Paloma S. (2015). Incentives and policies for integrated pest management in Europe: A review. Agron. Sustain. Dev..

[18] Clark B., Hillocks R., Pimentel D., Peshin R. (2014). Integrated pest management for European agriculture. Integrated Pest Management.

[19] Das G.P., Magallona E.D., Raman K.V., Adalla C.B. (1992). Effects of different components of IPM in the management of the potato tuber moth, in storage. Agric. Ecosyst. Environ..

[20] Simoni S., Castagnoli M., Ciancio A., Mukerji K.G. (2010). IPM strategies through specialist and generalist phytoseiids (Acari, Mesostigmata). Integrated Management of Arthropod Pests and Insect Borne Diseases.

[21] Van Lenteren J.C. (2012). The state of commercial augmentative biological control: Plenty of natural enemies, but a frustrating lack of uptake. BioControl.

[22] De Moraes G.J., Venancio R., dos Santos V.L., Paschoal A.D., Carrillo D., de Moraes G.J., Peña J.E. (2015). Potential of Ascidae, Blattisociidae and Melicharidae (Acari: Mesostigmata) as biological control agents of pest organisms. Prospects for Biological Control of Plant Feeding Mites and other Harmful Organisms.

[23] Haines C.P. (1981). Laboratory studies on the role of an egg predator, *Blattisocius tarsalis* (Acari: Ascidae), in relation to the natural control of *Ephestia cautella* (Lep.: Pyralidae) in warehouses. Bull. Entomol. Res..

[24] Nielsen P.S. (1999). The impact of temperature on activity and consumption rate of moth eggs by *Blattisocius tarsalis* (Acari: Ascidae). Exp. Appl. Acarol..

[25] Gallego J.R., Gamez M., Cabello T. (2019). Potential of the *Blattisocius mali* Mite (Acari: Blattisociidae) as a biological control agent of potato tubermoth (Lep.: Gelechiidae) in stored potatoes. Potato Res..

[26] Thomas H.Q., Zalom F.G., Nicola N.L. (2011). Laboratory studies of *Blattisocius keegani* (Acari: Ascidae) reared on eggs of navel orange worm: Potential for biological control. Bull. Entomol. Res..

[27] Barker P.S. (1967). Bionomics of *Blattisocius keegani* (Acarina: Ascidae), a predator on eggs of pests of stored grains. Can. J. Zool..

[28] Krantz G.W. (1998). Review reflections on the biology, morphology and ecology of the Macrochelidae. Exp. Appl. Acarol..

[29] Azevedo L.H., Emberson R.M., Esteca F.C.N., de Moraes G.J., Carrillo D., de Moraes G.J., Peña J.E. (2015). Macrochelid mites (Mesostigmata: Macrochelidae) as biological control agents. Prospects for Biological Control of Plant Feeding Mites and Other Harmful Organisms.

[30] Nesbitt H.J. (1951). A taxonomic study of the Phytoseiinae (family Laelaptidae) predaceous upon Tetranychidae of economic importance. Zool. Verh. Leiden.

[31] Haines C.P. (1978). A revision of the genus *Blattisocius* (Mesostigmata: Ascidae) with especial reference to *B. tarsalis* and the description of a new species. Acarologia.

[32] Greenspan L. (1977). Humidity fixed points of binary saturated aqueous solutions. J. Res. Natl. Bur. Stand..

[33] Fenemore P.G. (1977). Oviposition of potato tuber moth, *Phthorimaea operculella* (Lep.: Gelechiidae); fecundity in relation to mated state, age, and pupal weight. N. Z. J. Zool..

[34] Robertson J.L., Preisler H.K. (1992). Pesticide Bioassays with Arthropods.

[35] Nielsen P.S. (2003). Predation by *Blattisocius tarsalis* (Acari: Ascidae) on eggs of *Ephestia kuehniella* (Lep.: Pyralidae). J. Stored Prod. Res..

[36] Garcia-Martin M., Gamez M., Torres-Ruiz A., Cabello T. (2008). Functional response of *Chelonus oculator* (Hym.: Braconidae) to temperature, and its consequences to parasitism. Comm. Ecol..

[37] Juliano S., Scheiner S., Gurevitch J. (2001). Nonlinear curve fitting: Predation and functional response curves. Design and Analysis of Ecological Experiments.

[38] Hassell M.P. (1978). Arthropod Predator-Prey Systems.

[39] Cabello T., Gamez M., Varga Z. (2007). An improvement of the Holling type III functional response in entomophagous species model. J. Biol. Syst..

[40] Azevedo L.H., Ferreira M.P., de Campos Castilho R., Cançado P.H.D., de Moraes G.J. (2018). Potential of *Macrocheles* species (Acari: Mesostigmata: Macrochelidae) as control agents of harmful flies (Dip.) and biology of *Macrocheles embersoni* on *Stomoxys calcitrans* and *Musca domestica* (Dip.: Muscidae). Biol. Control.

[41] Messelink G., van Holstein-Saj R. (2008). Improving thrips control by the soil-dwelling predatory mite *Macrocheles robustulus*. IOBC WPRS Bull..

[42] Pozzebon A., Boaria A., Duso C. (2015). Single and combined releases of biological control agents against canopy-and soil-dwelling stages of *Frankliniella occidentalis* in cyclamen. BioControl.

[43] Filipponi A. (1964). The feasibility of mass producing macrochelid mites for field trials against houseflies. Bull. World Health Organ..

[44] Hassan M.F., Ali F.S., Hussein A.M., Mahgoub M.H. (2002). Biological studies on *Macrocheles muscaedomesticae* fed on different stages of potato tuber moth, *Phthorimaea operculella*. Egypt. J. Biol. Pest Control.

[45] Axtell R.C. (1991). Role of mesostigmatid mites in integrated fly control. Mod. Acarol..

[46] Trivedi T.P., Rajagopal D., Tandon P.L. (1994). Life table for establishment of potato tubermoth *Phthorimaea operculella*. J. Indian Potato Assoc..

[47] CABI Centre for Agricultural Bioscience International. *Phthorimaea operculella* (potato tuber moth). http://www.cabi.org/isc/datasheet/40686.

[48] Darst P.H., King E.W. (1969). Biology of *Melichares tarsalis* in association with *Plodia interpunctella*. Ann. Entomol. Soc. Am..

[49] Riudavets J., Maya M., Monserrat M. (2002). Predation by *Blattisocius tarsalis* (Acari: Ascidae) on stored product pests. IOBC WPRS Bull..

[50] Lindquist E.E., Krantz G.W., Walter D.E., Krantz G.W., Walter D.E. (2009). Order Mesostigmata. A Manual of Acarology.

[51] Treat A.E., Daniel M., Rosicky B. (1973). Association of the mite *Blattisocius tarsalis* with the moth *Epizeuxis aemula*. Proceedings of the 3rd International Congress of Acarology.

[52] Nielsen P.S. (2001). Developmental time of *Blattisocius tarsalis* (Acari: Ascidae) at different temperatures. Exp. Appl. Acarol..

[53] Thind B.B., Ford H.L. (2006). Laboratory studies on the use of two new arenas to evaluate the impact of the predatory mites *Blattisocius tarsalis* and *Cheyletus eruditus* on residual populations of the stored product mite *Acarus siro*. Exp. Appl. Acarol..

[54] Hajek A.E., Eilenberg J. (2018). Natural Enemies: An Introduction to Biological Control.

